# Peri-Interventional Prophylactic Plasma Transfusion

**DOI:** 10.3390/medicina62101901

**Published:** 2026-10-01

**Authors:** Daniela Filipescu, Cornelia-Elena Predoi, Cosmin Balan, Radu Filipescu, Dragos Guz, Cornelia Margineanu, Cornel Robu, Serban-Ion Bubenek-Turconi, Mihai Gabriel Ștefan

**Affiliations:** 1 Emergency Institute of Cardiovascular Disease “Prof. Dr. CC Iliescu”, 022322 Bucharest, Romania; 1danielaf@gmail.com (D.F.); raduzfil@gmail.com (R.F.); guz.dragos@gmail.com (D.G.);; 2 Faculty of Medicine, University of Medicine and Pharmacy “Carol Davila”, 020021 Bucharest, Romania

**Keywords:** plasma transfusion, fresh frozen plasma, patient blood management, coagulation tests, international normalised ratio, invasive procedures, anticoagulant reversal, perioperative bleeding

## Abstract

Human plasma remains one of the most frequently transfused blood products despite increasing data that many transfusions are unnecessary and unsupported by clinical evidence. This review summarises current evidence regarding the haemostatic properties of therapeutic plasma, their effects on conventional coagulation tests, and contemporary use in non-bleeding patients undergoing surgery or invasive procedures, as well as for anticoagulant reversal. Conventional coagulation tests, particularly the international normalised ratio (INR), correlate poorly with bleeding risk and are only minimally corrected by commonly administered plasma doses. Current evidence demonstrates that prophylactic plasma transfusion does not reduce peri-procedural bleeding in most patients while exposing them to transfusion-related complications which can lead to worse outcomes. Recent international guidelines consistently discourage routine plasma transfusion solely to correct mildly abnormal coagulation tests, and recommend four-factor prothrombin complex concentrate rather than plasma for urgent vitamin K antagonist reversal.

## 1. Introduction

Human plasma is one the most frequently transfused labile blood products [1]. Unfortunately, in many cases, plasma transfusion is inappropriate [2,3,4], contributing to the waste of a precious and scarce medical resource, represented by human blood [5]. Despite its use to correct haemostatic disorders in bleeding and non-bleeding patients for decades, the evidence for the efficacy of plasma is inconsistent and low [6]. In particular, the role of prophylactic plasma transfusion in correcting abnormalities of conventional coagulation tests (CCTs) in non-bleeding patients is not supported, while it exposes the recipients to the risk of multiple adverse effects, which include transfusion-associated circulatory overload (TACO), transfusion-related lung injury (TRALI), febrile reactions, allergic reactions, citrate toxicity, haemolytic reactions, transfusion-associated immunomodulation, and graft-versus-host disease, infections, and multiorgan failures [6,7].

This review discusses the evidence and most recent recommendations regarding the prophylactic use of plasma to correct haemostatic abnormalities or reverse anticoagulants in non-bleeding patients undergoing surgical or invasive procedures. The use of plasma for the management of active bleeding is beyond the scope of this review. This is a narrative review. It was not conducted or registered as a systematic review, and no formal quantitative synthesis or risk-of-bias assessment was undertaken; the literature search and selection process are described in the following section. The aim is to reduce non-evidence-based plasma transfusion across peri-interventional practice in different specialties.

## 2. Materials and Methods

This narrative review is based on a literature search of PubMed/MEDLINE, Embase, and the Cochrane Library from inception to 1 June 2026, using combinations of the terms “plasma transfusion”, “fresh frozen plasma”, “prophylactic transfusion”, “coagulation tests”, “international normalized ratio”, “prothrombin time”, “activated partial thromboplastin time”, “invasive procedures”, “perioperative bleeding”, “vitamin K antagonist”, “prothrombin complex concentrate”, and “patient blood management”, combined using the Boolean operators AND and OR. The search was restricted to studies in adult humans published in English. Randomised controlled trials, systematic reviews and meta-analyses, observational cohort studies, audits of transfusion practice, and current clinical practice guidelines were considered eligible; case reports, conference abstracts, and studies restricted to paediatric populations were excluded. Studies addressing plasma transfusion in actively bleeding patients were excluded unless they informed the haemostatic properties of plasma preparations. Given the heterogeneity of study designs, populations, and outcome definitions, the findings were synthesised narratively; no formal quantitative synthesis or risk-of-bias assessment was undertaken.

## 3. Therapeutic Plasma Preparations

The majority of therapeutic plasma used worldwide consists of fresh frozen plasma (FFP; strictly, fresh plasma preserved by rapid freezing—the conventional term is retained here for consistency with the cited guidelines and nomenclature) obtained from single-donor plasma. Other types, which differ by the production process or the method of virus inactivation, are also available. Single-donor plasma is obtained by separating plasma from whole blood donations through centrifugation or by apheresis collection, rapidly frozen within 8 h of collection for FFP, or between 8 and 24 h for PF-24, and stored at ≤−25 °C, below the eutectic point of plasma of approximately −23 °C (the FDA and AABB standard of ≤−18 °C, still applied in the United States, lies above this point) and stored over a quarantine period of time during which the donors should remain negative for different viruses [1,6,8]. Besides routine microbiological testing, different pathogen reduction methods may be applied. Those in current clinical use for plasma are photochemical treatment with amotosalen and ultraviolet A light, methylene blue with visible light, and the solvent/detergent process. The first two are applied to single-donor units, the photoactive compound being removed by absorption filtration before freezing, whereas the solvent/detergent process is applied to pooled plasma [1,7], although they may decrease the haemostatic capacity of the processed plasma (7).

For administration, FFP should be thawed and transfused within 4–6 h [1,7]. In many European countries, a 24 h period is the standard for thawed plasma storage, because of concerns over reduced efficacy as a consequence of decreases in coagulation factor levels [9], but in United States (US), it is allowed to store thawed FFP and PF-24 at 1–6 °C for 5 days if the plasma was prepared in a closed system [7,8,10].

The solvent detergent plasma (SDP) is pooled from hundreds of blood group-compatible single donors, which undergoes solvent detergent procedure to eliminate lipid-coated viruses before it is frozen [1]. The majority of the coagulation factors present in SDP are reduced but, due to the wide reference range of factors in the single-donor units, the decrease in their activity levels is not clinically significant [7].

Liquid plasma is a single-donor plasma which has never been frozen but stored at 1–6 °C for up to 5 days after being removed from a whole blood unit before its expiration time [6,7,9]. As liquid plasma contains reduced levels of fibrinogen, factors V and VIII, and von Willebrand (vW) multimers, it has been used mostly in plasmapheresis and less in bleeding situations [8].

Lyophilised or freeze-dried human plasma originates from filtrated and frozen single-donor plasma from which the liquid has been removed under vacuum pressure [11]. It is stored at ambient temperature and reconstituted in sterile water immediately before administration [1,6]. The main characteristics of plasma preparations are presented in Table 1.

The panel of quality criteria for plasma preparations usually includes pH, osmolality, total protein content, absence of irregular red blood cell (RBC) antibodies, hepatitis A virus antibody levels, and coagulation factor concentration ranges, but the criteria may vary from country to country [9]. For haematological quality, it is recommended that RBCs are <6 × 10^9^/L, thrombocytes < 50 × 10^9^/L, and white blood cells < 0.1 × 10^9^/L, although leukoreduction is not universally requested [1].

## 4. Haemostatic Capacity of Plasma

Plasma contains physiological levels of all coagulation factors and inhibitors, including antithrombin, protein C and S, and tissue factor inhibitor, and an anticoagulant solution added during the collection [6]. By convention, plasma from a healthy donor contains approximately one international unit (IU) of each coagulation factor per millilitre, so a 250 mL unit would be expected to contain approximately 250 IU of each factor. Dilution by the anticoagulant–preservative solution added at collection (e.g., CPD: citrate, phosphate and dextrose) reduces this to approximately 200 IU [12]. This figure therefore expresses the total quantity of coagulation factor activity delivered by a unit of plasma, and is not a separately measured laboratory parameter. The concentration of fibrinogen is 2–4 mg/mL or, depending on the volume of the unit, approximately 400–900 mg [1]. The plasma concentrations of coagulation factors are sufficient for physiological conditions but may be insufficient for correction of different coagulopathies, and the presence of endogenous and added anticoagulants further impede the haemostatic effect [6].

Generally, the activities of coagulation factors in plasma preparations vary substantially depending on the individual donor’s coagulation factor level, which is influenced by age, blood group, and smoking status of the donor, the pathogen inactivation, and thawing processes [1,6]. The losses are not uniform: they affect principally the labile factors, factor VIII, fibrinogen, and factor XI falling by approximately 30–40% with pathogen inactivation (see Section 3), while the remaining factors are largely preserved [13]. The acute-phase proteins fibrinogen and factor VIII (FVIII) show particularly wide variation. As a quality standard criterion, plasma should have a FVIII activity > 70% or at least 0.7 IU/mL of FVIII in at least 75% of units immediately after thawing before distribution [6], but more coagulation factor criteria may be in place in different countries [9].

The concept of a single haemostatic threshold is a simplification, since the relationship between the activity of an individual coagulation factor and the clinical bleeding phenotype differs considerably between factors. Most coagulation factors provide adequate haemostasis at activities of approximately 25–30%, and this is the level generally targeted when plasma is transfused [7]. Evidence from inherited deficiencies shows, however, that this figure cannot be applied uniformly. In congenital factor V deficiency, bleeding severity correlates with activities below 0.1 IU/mL, whereas heterozygous carriers with activities of 0.2–0.6 IU/mL are asymptomatic or bleed only mildly, and substitution is directed at maintaining levels of merely 15–20% [1]. In congenital factor XI deficiency, the correlation is weaker still: severe bleeding has been reported at activities of 0.09–0.41 IU/mL while non-bleeding patients have had activities of 0.14–0.39 IU/mL, ranges that overlap almost entirely, so that factor XI activity does not distinguish patients at high from those at low bleeding risk before invasive procedures [14]. The conventional coagulation tests reflect the same heterogeneity, since prolongation of the PT requires the deficient factor to fall below approximately 30% activity, and prolongation of the aPTT requires activities below 15–35%, depending on which factor is affected [7].

Two implications follow. First, an abnormal coagulation test result cannot be translated into a uniform statement about haemostatic competence, since the same degree of prolongation may reflect different combinations of factor deficiencies with different bleeding implications. Second, and more relevant to the present review, thresholds derived from patients with a single congenital deficiency, and otherwise intact haemostasis cannot be transferred to acquired multifactorial coagulopathy, in which procoagulant factors, natural anticoagulants, and fibrinolytic proteins are reduced in parallel and the net haemostatic balance may be preserved.

Although different plasma preparations are considered having equivalent haemostatic effects [1], due to the variability in physiological content of human plasma, amplified by the processes applied to obtain the final product, the haemostatic capacity of plasma preparations may also differ [9,15].

The recommended dose of plasma is 10–15 mL/kg, which in an average adult equals approximately four units or 1000 mL of plasma [1]. Doses below 600 mL or two to three units are considered inadequate in adults [6]. Moreover, guidelines on management of bleeding suggest even higher doses of plasma, of 15–20 mL/kg [16]. However, the presence of impaired renal function, severe liver damage, or cardiopulmonary insufficiency, limits the plasma dose due to the risk of TACO.

The recommended dose of plasma is expected to increase the clotting factor levels by about 10–20% from baseline [8,12]. This was confirmed in a randomised controlled trial (RCT) conducted in non-bleeding critically ill patients with an INR of 1.5–3.0 scheduled for bedside invasive procedures, which found an increase of factor activities by 10–12% after infusion of 12 mL/kg of FFP [17]. In contrast, a retrospective study of 30 surgical intensive care unit patients with ongoing coagulopathic bleeding, approximately half of whom had sustained trauma, found that the volume of FFP required to normalise a prolonged thromboelastographic reaction time was considerably lower than the recommended dose, averaging 6.8 mL/kg in high responders and corresponding to approximately two units. The authors calculated that this volume would raise coagulation factor concentrations by 15.6%, and emphasised the importance of the recipient’s initial plasma volume in estimating the amount of FFP required [18].

However, it remains unclear which level of coagulation factor activity would be needed to secure haemostasis in both bleeding and non-bleeding patients. Usually, the goal of plasma transfusion is to supplement or replace deficient factors to reach levels > 30% for effective haemostasis [3,8]. Due to the donor-dependent activity of coagulation factors and presence of inhibitors in plasma, a clinical and significant increase of coagulation factor activities was thought to be achieved by higher doses of plasma. A small study performed in critically ill patients that compared doses of 12.2 mL/kg versus 33.5 mL/kg found significant increases in coagulation factor activity (about 25–35%) with the higher dose only, albeit with substantial differences for the individual factors [19]. The authors acknowledged the low clinical applicability of the 30 mL/kg dose (equivalent to 10 U of FFP in an average adult of 80 kg) and did not support a specific weight-related dose of plasma to all patients but a tailored, individualised approach. Moreover, the adverse effects of plasma transfusion are dose-dependent, which counterbalance the potential benefits of higher doses [20].

### 4.1. Effect of Plasma on Coagulation Tests

Since the goal of plasma transfusion is to supplement deficient coagulations factors, it is important to confirm the severity of the deficiency and monitor the effectiveness of the intervention. The CCTs most utilised to determine the necessity of plasma transfusion are the prothrombin time (PT), international normalized ratio (INR), and the activated partial thromboplastin time (aPTT). Prolongations of PT and aPTT indicate activity of factors VII, X, V, II, and/or fibrinogen, and of factors XII, XI, IX, and VIII, respectively, below 15–35%, depending on the factor affected [7]. The discussion that follows concentrates on the PT/INR because it is the test that triggers most prophylactic plasma transfusions in practice and the laboratory endpoint used by nearly all studies of plasma efficacy. This reflects the literature rather than any view that the INR is the appropriate measure; the limitations of the aPTT and the potential role of global assays are addressed in Section 4.2 and Section 4.3.

The INR allows for comparison of PTs between laboratories by accounting for differences in reagents and instrumentation and, if it is prolonged, it indicates that one of the vitamin K-dependent coagulation factors (II, VII, IX, X) is below the haemostatic threshold. Due to wide availability, INR is also often used to assess bleeding risk and the effectiveness of FFP transfusion, even though the test was designed to monitor therapy with vitamin K antagonists (AVKs) and not to trigger administration of FFP in clinical practice [21]. Indeed, the INR represents only a part of the coagulation cascade, it is insensitive to the activities or concentrations of endogenous anticoagulant and fibrinolytic proteins, and is not a reliable indicator of the complexity of coagulopathies [22].

The PT is measured by incubating citrated plasma with tissue factor and recording the time to clot formation after recalcification; it evaluates the extrinsic and common pathways, so that prolongation indicates reduced levels or function of factors VII, X, V, II, and/or fibrinogen. The INR is calculated from the patient’s PT, the mean normal PT of the laboratory, and the international sensitivity index of the reagent–instrument combination, and was introduced to allow comparison between laboratories rather than as a marker of bleeding risk. The aPTT is measured after incubation with phospholipid and a contact activator, and evaluates the intrinsic and common pathways, adding factors XII, XI, IX, and VIII. Both tests are insensitive to mild deficiencies: the PT is prolonged only when the affected factor falls below approximately 30% activity, and the aPTT only below 15–35%, depending on the factor [7].

The pattern of abnormality is informative. Isolated prolongation of the PT/INR suggests factor VII deficiency or an early deficiency of the vitamin K-dependent factors, as in vitamin K antagonist therapy, vitamin K deficiency, or hepatic dysfunction. Isolated prolongation of the aPTT with a normal PT is the characteristic pattern of haemophilia A and B [14], and also occurs with deficiency of factor XI or XII, heparin exposure, or a lupus anticoagulant. Prolongation of both tests suggests deficiency of the common pathway factors or fibrinogen, established liver disease, disseminated intravascular coagulation, or dilutional coagulopathy. The distinction matters because in several of these situations plasma is ineffective or inappropriate, anticoagulant reversal or a specific factor concentrate being preferable [7].

Normal haemostasis occurs when coagulation factor concentrations are generally more than 30%, which correlates with an INR of approximately 1.7 [23]; at INR of 3, the coagulation factor concentrations are less than 10% of normal [12,22]. These correspondences should be interpreted with caution. They were derived largely in patients receiving vitamin K antagonists, in whom the vitamin K-dependent factors decline together, and they are not fixed. The INR is calculated using the international sensitivity index of the particular reagent and instrument combination, so the same factor activities may yield different values between laboratories, and the relationship between a given INR and the underlying factor levels varies with the clinical condition and with which factors are reduced [7]. In acquired multifactorial coagulopathy, and particularly in liver disease, the conventional tests do not reflect the overall haemostatic balance and their abnormalities must be interpreted accordingly [13,24]. Despite an elevated INR, patients may not necessarily have an increased bleeding tendency, as sufficient clotting activity may be present at INR > 2.5 [21]. It results that coagulation tests are poor predictors of the actual level of coagulation factors and bleeding risk [19].

However, FFP was often used to correct haemostatic abnormalities that have been identified on CCTs in non-bleeding patients who are about to undergo an invasive procedure or considered at risk of bleeding. Although the effect of plasma transfusion on INR reversal was the focus of research in transfusion medicine over two decades ago, there seems to have been little knowledge transfer to other specialties [12]. It is important to acknowledge that the inaccurate picture of coagulation status and poor correlation of coagulation tests changes with actual coagulation factor content was signalled by haematologists more than 20 years ago [25].

The magnitude of the effect that plasma can have on the INR is determined chiefly by the pre-transfusion value. The effect of plasma transfusion on the INR was modelled in 2006, when it was demonstrated that at a low starting INR, the effect of plasma transfusion is minimal, that more than two units of plasma is required to correct a high INR into a more normal range, and that the higher the patient’s starting INR, the greater the effect of plasma transfusion had in lowering it, whereas, even after transfusing multiple units of plasma, the INR never really reached 1.0 [26]. It was also shown that fewer than 1% of patients with a mild increase (up to 1.85) had a normalisation of their INR after plasma transfusion [27]. One explanation for these facts relates to the INR of the transfused plasma which reflects the INR of the donor, typically ranging from 0.9 to 1.3, values close to mild abnormalities [12]. Unfortunately, many clinicians still order plasma in two-unit doses expecting to see reversal of a patient’s mild coagulopathy [3,4,28] but obtain nothing but exposing the patient to the risks of transfusion-associated complications [12].

This is not a historical concern. Contemporary audits show that approximately a third of plasma transfusion events are triggered by a pre-transfusion INR below 1.8, and that a quarter to a third of all plasma is administered to non-bleeding patients, frequently at an INR below 1.5 [3,4]. These data are presented in Section 5.1.

Another reason for the small effect of plasma on low INR values is the nonlinear relationship between coagulation factor concentration and INR [12,26]. Transfusion of plasma to raise a patient’s coagulation factor concentration by 10% would drop an INR of 3 to approximately 2.1, but change an INR of 1.7 very little, slightly down to 1.6, suggesting that attempts to correct small elevations in the INR are futile [12] (Figure 1). Accordingly, plasma transfusions for INR of less than 1.6–1.7 and doses of one to two units of plasma are currently strongly discouraged [6].

A more global assessment of haemostasis with VETs or thrombin generation potential would be more useful to assess the haemostatic capacity of plasma. However, in a study in non-bleeding coagulopathic critically ill patients having prolonged INRs, reduced levels of individual coagulation factors, and a tendency towards hypocoagulability as measured by whole blood assays, FFP (12 mL/kg) failed to alter the haemostatic balance to a more procoagulant state, showing marginal effects on thrombin generation and thromboelastometry variables despite an increase in individual factor levels [29].

A validated model predicting the post-transfusion INR from the pre-transfusion value, the volume transfused, and the patient’s characteristics reproduces these observations [30].

The correction achievable with plasma depends far more on the pre-transfusion INR than on the dose administered. The relationship between plasma administration and absolute correction in INR values was studied in a cohort of 6779 patients with varying levels of coagulation abnormalities and comorbidities and in a variety of clinical settings [28]. The majority (85%) of transfusions occurred perioperatively, with 20% of transfusions administered prophylactically before a procedure. The median pre-transfusion INR was 1.9 and the median decreases in INR and PT were 0.4 and 3.7 s, respectively. Only 12% and 61% of patients achieved complete (i.e., INR ≤ 1.1) or partial INR normalisation (i.e., INR ≤ 1.5) after plasma transfusion, respectively. Reductions in INR were modest with pre-transfusion INR values < 3. In a post-hoc analysis, the median decrease in INR after plasma transfusion in those with INR > 3.5 was 2.4 compared to a median decrease of 0.3 in those with INR < 2.5. The dose had far less influence than the starting value (Table 2) [28].

Similarly, FFP transfusion had only a marginally beneficial effect on improving coagulation profiles in a RCT performed in critically ill patients with prolonged INR (1.5–3.0) undergoing bedside invasive procedures [17]. INR improved in only 54% of the plasma transfused patients. Although levels of FII, FV, and FVII increased, anticoagulant factor levels were also elevated, and thrombin generation was unaffected [17]. Furthermore, in a sub-study on sepsis patients of a large international analysis of transfusion practice in intensive care units (ICUs), pre-transfusion INR (median 1.6) decreased by only 0.2 following plasma [32].

The evidence considered so far concerns the effect of plasma on laboratory parameters. Whether that effect translates into clinical benefit is a separate question, and one on which the data point in the opposite direction. It can be argued that the minimal effect of FFP transfusion on INR seen in different studies is due to low doses used, but there is no substantial evidence that higher plasma volumes result in improved clinical outcomes. In a study including 3393 patients receiving intraoperative median plasma transfusion of two units, greater improvements in INR were associated with improved outcomes, but higher plasma volumes were associated with worse outcomes, increased odds for intraoperative and postoperative RBC transfusion, and fewer hospital- and ICU-free days registered for each 1 mL/kg increase in plasma administered [31]. The authors attributed this to the haemodilution being inadvertently induced by a higher dose of plasma, potentially decreasing haemoglobin values beyond the threshold for transfusion.

### 4.2. The Role of aPTT

The aPTT has been investigated far less than the INR as a trigger for plasma transfusion, and the available evidence does not support its use in this role. As a predictor of procedural bleeding, it performs no better than the PT/INR; the evidence-based reviews that found no relationship between abnormal coagulation test results and bleeding after invasive procedures, having examined both tests [22,23]. In a nationwide cohort study, the risk of spinal haematoma after lumbar puncture was similar in patients with and without coagulopathy, the latter defined to include an aPTT above 39 s [40]. Current guidance accordingly recommends against routine coagulation screening before invasive procedures, on the explicit grounds that it does not indicate bleeding risk, and against the routine use of plasma to correct abnormal results, advising a structured bleeding history instead [41].

Conventional triggers, such as a PT or aPTT prolonged beyond 1.5 times the upper limit of the reference range [13,42], therefore rest on convention rather than on demonstrated predictive value. Interpretation is further limited by the insensitivity of the test, which is prolonged only once the affected factor falls below 15–35% activity [7], and by the observation that in critically ill patients the aPTT and PT are frequently prolonged without individual coagulation factors being reduced to a degree that compromises haemostasis [19]. An isolated prolonged aPTT in a non-bleeding patient more often reflects heparin exposure or an antiphospholipid antibody than a haemostatic defect, and in neither case is plasma effective; such a finding warrants a mixing study and specific factor assays rather than empirical transfusion [7].

As a measure of the effectiveness of transfusion, the aPTT is subject to the same constraint as the INR, since the aPTT of the transfused plasma itself lies within the normal range, so that the achievable correction is bounded and, at mildly abnormal starting values, negligible.

### 4.3. Viscoelastic Testing

Viscoelastic tests provide a more global assessment of haemostasis than the conventional coagulation tests, but their established role lies in the algorithmic management of active bleeding, where they reduce plasma transfusion without increasing bleeding and are recommended by current guidelines [42,43]. In the prophylactic setting the evidence is limited. Viscoelastic parameters are relatively insensitive to the moderate reductions in factor activity that produce a mildly prolonged INR and correlate poorly with the conventional tests [18], and in non-bleeding coagulopathic critically ill patients, FFP produced only marginal changes in thromboelastometry variables and thrombin generation, despite measurable increases in factor levels [29]. Where viscoelastic testing has been compared with conventional testing before an invasive procedure, it identified patients in whom transfusion could safely be withheld rather than those who benefited from it: in a randomised comparison of restrictive, thromboelastometry-guided and conventional test-guided protocols before central venous catheterisation in cirrhosis, bleeding did not differ between strategies and the restrictive approach most reduced transfusion and costs [34]. Viscoelastic testing therefore currently supports the avoidance of prophylactic plasma rather than its direction, and whether it can guide prophylaxis to improve outcomes remains a research priority [44].

## 5. Prophylactic Use of Plasma

Plasma was used to prevent and treat coagulopathy caused by loss or consumption of coagulation factors in different bleeding and non-bleeding situations, including reversal of AVK anticoagulation and to treat single factor deficiencies associated or not with blood losses. However, the prophylactic use of plasma transfusion is not supported by robust data for efficacy and is associated with risks of adverse transfusion reactions [6]. Besides the known complications, plasma transfusion, in addition to RBCs, increased the incidence of venous thrombotic events, pulmonary embolisation, and mortality in surgical patients without proven coagulopathy [45]. Considering the large evidence for both inadequate dosing and unnecessary use, the current indications for prophylactic plasma transfusion are rather restrictive [44].

### 5.1. Prophylactic Plasma Transfusion Before Surgery and Invasive Procedures

Prophylactic FFP to correct abnormal coagulation tests before invasive procedures, including surgery, is common with the majority of transfusion episodes occurring in those without active bleeding [3,4,28]. Although not supported by evidence [46], this indication is based on the assumptions that an elevated INR confers increased risk of bleeding, that transfusion of plasma will supplement the insufficient coagulation factors to the desired level of correction of INR, and that INR correction decreases the incidence of bleeding [28].

The lack of the INR’s predictive ability has been demonstrated in numerous studies and was the focus of a systematic review which showed no relationship between abnormal INR values and procedural bleeding [22]. The minimal effect of plasma on INR at low values of the test has been described above. The impact of commonly used doses of FFP to reduce the bleeding risk is very limited, particularly when INR is between 1.5 and 1.9 [13]. As for the lack of clinical benefit of prophylactic plasma, evidence continues to accumulate.

Prophylactic plasma has not been shown to reduce bleeding in any of the populations in which it has been studied. A RCT conducted in critically ill patients with prolonged INR (1.5–3.0) undergoing invasive procedures did not find differences in bleeding complications regardless of whether prophylactic FFP (12 mL/kg) was administered or not [17]. Similarly, a large retrospective ICU cohort study found that prophylactic FFP transfusion did not improve patient outcome and was paradoxically associated with increased RBC requirements [35]. The higher rate of perioperative and intraoperative RBC transfusion, as well as an increased rate of reoperation for bleeding, was also reported in non-cardiac surgery patients transfused preoperatively with plasma for an INR ≥ 1.5 [36]. These findings were partially explained by the increased haemodilution induced by plasma transfusion [35] and the anticoagulant content of plasma [36].

However, a Cochrane database systematic review on prophylactic use of FFP undergoing non-cardiac surgery or invasive procedures could neither support nor oppose it because of limited RCT evidence [39]. Consequently, clinicians continued to administer plasma prophylactically, despite the risk of harm associated with transfusion and in contrast to guidelines already published around the world since 2010 which advised against pre-procedure and prophylactic plasma in non-bleeding patients with abnormal coagulation tests [6,13,47].

Audits of contemporary practice show that plasma continues to be given prophylactically at coagulation values at which it can have no meaningful effect. In a recent prospective international ICU cohort performed in 233 centres in 30 countries and on six continents that included 3643 patients, 10% received plasma (547 transfusion events with a median of two units per event) [4]. Over a third of patients receiving plasma did not have bleeding (prophylactic use 25% and pre-procedure 12%). Target INR/PT was not stated in more than half of transfusions and pre-transfusion INR/PT or VET were not reported for 27% of events. In non-bleeding transfusion events, INR was <3 and <1.5 in 54% and 30% of cases, respectively. Similarly, in other cohort studies, the pre-procedure indication for prophylactic plasma was reported in up to 27% of cases [48,49]. Furthermore, a large audit which evaluated 46,377 units of plasma transfused in 11,163 admissions across 23 hospitals in Canada also found high variability and suboptimal transfusion practices across sites and physician specialties [3]. Median pre-transfusion INR and the mean number of plasma units transfused per event and by site ranged from 1.5 to 2.5 and from 1.0 to 3.0, respectively. The majority of plasma transfusion dose was sub-therapeutic. In the entire cohort, 35.5% of plasma transfusion events were for a pre-transfusion INR < 1.8, and at eight sites, at least 20% of the events were triggered by a pre-transfusion INR < 1.5, despite the Choosing Wisely national campaign’s recommendation against this practice. Notably, in 5078 plasma events without a reported pre-transfusion INR, 48% and 63% of plasma transfusions were not preceded or followed by RBC transfusions, respectively, suggesting a non-bleeding indication of plasma. These results highlight the need of structured education of professionals to optimise guideline implementation.

As the efficacy of prophylactic plasma for the reduction of peri-interventional bleeding remains unproven and because of the widespread inappropriate use of plasma for this purpose, new international guidelines specifically advise against the routine prophylactic use of FFP to correct abnormal coagulation results before invasive procedures in non-bleeding patients [41,42,47,50]. Procedures are conventionally stratified by bleeding risk rather than by surgical magnitude, the risk being determined by the compressibility of the site and the consequence of bleeding into it rather than by the extent of the intervention; the classification in current use is summarised in Table 3 [24]. The most recent guidelines from the American College of Chest Physicians favour a first line no-transfusion approach in procedural settings and suggest (conditional recommendations based on very low certainty of evidence) against routine prophylactic FFP in critically ill patients at increased risk of coagulopathy for vascular procedures (central venous catheter or arterial line insertion), bedside thoracentesis, paracentesis or flexible bronchoscopy and gastrointestinal endoscopy without biopsy [44]. Transfusion of plasma is considered only in patients at high risk of bleeding, or where the bleeding complication may be catastrophic (e.g, lumbar puncture). In the latter case, the suggestion is to keep INR between 1.5 and 2 [44], despite the results of a Cochrane analysis which found no evidence from RCTs and observational studies to support or refute plasma transfusions prior to insertion of a lumbar puncture needle or epidural catheter, or what would be the degree of coagulopathy requiring plasma transfusion [51]. The positions of the principal guidelines and consensus documents on pre-procedural coagulation test thresholds are summarised in Table 4. With the single exception of the conditional suggestion for lumbar puncture, no document specifies a coagulation test value at which prophylactic plasma is indicated in a non-bleeding patient, and no recommendation in this area rests on more than low certainty evidence.

Moreover, a large Danish database study showed that the risk of spinal haematoma after lumbar puncture in patients with or without coagulopathy (platelet counts < 150 × 10^9^/L, INR > 1.4 and/or aPTT > 39 s) was similar (0.2%), irrespective of the severity of the coagulation abnormality [40].

A risk/benefit assessment should be made prior to each FFP transfusion and, given the known risks of blood product transfusion and the limited data regarding the benefits from plasma, most patients would benefit from avoiding prophylactic transfusion [44]. Future research is needed to explore VETs to guide transfusions, alternative diagnostic modalities, and image guidance in high bleeding risk procedures or procedures with severe bleeding-associated risk, such as lumbar punctures [44].

#### 5.1.1. Severe Liver Disease

Patients with severe liver disease have complex disturbances of the coagulation system, consisting of concomitant reduced activities of coagulation factors, thrombocytopenia, and decreased inhibitors of fibrinolysis as well as decreased activity of inhibitors of the coagulation system, supporting the concept of a “re-balanced haemostasis” on a lower activity level in these patients, which correlates with clinical observations of a normal bleeding tendency and no increased transfusion requirements even in high risk surgeries [1]. This complex pathophysiology is not reflected by the CCTs (PT, INR, aPTT) that often are prolonged and may suggest hypocoagulability and the need of haemostatic products before interventional procedures or surgery, although these will probably not be needed [1,50,54].

In a small pilot trial in hospitalised cirrhotic patients with INR 1.5–2.5 undergoing procedures conducted outside the operating room, randomly allocated to receive plasma transfusion (10–15 mL/kg) or no transfusion, the change in INR was small (mean −0.24), corrected to <1.5 in a minority of patients (38.5%) in the plasma arm, and not associated with haemoglobin changes [33]. Similarly, a small RCT study in critically ill cirrhotic patients comparing three transfusion protocols (restrictive, thromboelastometry-, and CCT-based) prior to central venous catheterisation, found no difference in bleeding, but the restrictive strategy significantly reduced blood transfusion and costs [34].

Moreover, in cirrhotic patients, plasma transfusion may enhance an existing prothrombotic state [24,55]. Notably, ex vivo research pointed to the increased thrombin generation associated with prophylactic FFP and platelets and potentially increased risk of thrombosis [56]. In contrast, others found both enhanced and decreased thrombin generation after FFP transfusion in cirrhotic patients and speculated that FFP transfusion could decrease thrombin generation by replenishing protein C [57].

The detrimental effect of FFP in cirrhotic patients was further confirmed in a large cohort study of patients with decompensated cirrhosis with significant coagulopathy, which found no improvement in coagulopathy and even an association between FFP transfusion and FFP volume with increased risk of liver failure and respiratory failure events [38].

Considering the pathophysiological background and the clinical evidence, guidelines from different organisations agree and support a restrictive approach to correct abnormal coagulation tests with plasma or other haemostatic agents in patients with stable, i.e., nonbleeding, liver failure [13,24,41,42,50,52,53]. The only exception from this is the placement of an external ventricular drainage for intracranial pressure monitoring in acute liver failure [42]. On the other hand, invasive procedures may be replaced by alternative procedures with a lower bleeding risk (e.g., transjugular instead of percutaneous liver biopsy) and vitamin K should be administered in patients with prolonged PT that is likely to be due to acquired vitamin K deficiency [13]. This warrants explanation, since vitamin K cannot restore hepatic synthetic capacity. A prolonged PT in liver disease reflects both reduced hepatic synthesis of coagulation factors, which vitamin K does not address, and a coexisting functional deficiency of vitamin K, which it does. Vitamin K is fat-soluble, so cholestasis impairs its absorption, while poor dietary intake and prolonged antibiotic therapy contribute further; these circumstances are common in cirrhotic patients admitted with acute illness, although true deficiency is rare in stable ambulatory disease; since the two mechanisms cannot be distinguished from the PT alone, and vitamin K is inexpensive and low-risk, a trial of a single 10 mg dose is reasonable, with repeated dosing not recommended if the PT/INR is unchanged at 12–24 h [24].

#### 5.1.2. Cardiac Surgery

Plasma was frequently used for bleeding prophylaxis after cardiac surgery involving cardiopulmonary bypass (CPB), despite no evidence to support this practice. A meta-analysis of 15 RCTs (14 trials on prophylactic use), including 755 patients undergoing cardiac surgery, who received prophylactic FFP found no evidence of decreased perioperative blood but a significant increase in the number of patients requiring RBC transfusion who were randomised to FFP [37]. In light of these results, current European and US guidelines do not recommend the prophylactic use of FFP in elective cardiac surgery with CPB [42,43,50,58,59].

## 6. Reversal of Anticoagulants

A therapeutic INR for a patient on AVK is between 2 and 3.5, depending on the drug and indication for anticoagulation [7]. When the INR value exceeds the therapeutic targets, usually there is no need to antagonise the anticoagulant in the absence of bleeding. Importantly, most factor levels remain >30% until INR > 2.5 [21,28]. If the patient is not bleeding, even at INR > 10, only oral vitamin K supplementation is recommended, but in case of active bleeding, replacement of vitamin K dependent factors is needed [7,60]. However, in patients on AVK needing emergency surgery, the INR levels triggering reversal therapy are much lower. For example, an INR of <1.6 has been established as a safe target in patients who need hip fracture surgery [61].

Plasma and four-factor (4F) prothrombin complex concentrate (PCC) are the two products of choice to reverse AVKs effects, as they both contain the vitamin K-dependent factors in adequate levels when administered in proper doses. PCC preparations are not interchangeable. All non-activated PCC products contain factors II, IX, and X, but they differ in their factor VII content: three-factor products provide low concentrations of factor VII relative to the other factors, whereas four-factor products contain higher concentrations, and also include the anticoagulant proteins C and S [7,62]. Since the INR is most sensitive to factor VII, the absence of adequate replacement of this factor accounts for the weaker INR response to three-factor PCC. In a systematic review and meta-analysis of nine studies including 937 patients, four-factor PCC conferred more than three times the odds of reversal to the goal INR compared with three-factor PCC (odds ratio 3.61, 95% CI 1.97–6.60), with no significant difference in thromboembolic events (odds ratio 1.56, 95% CI 0.83–2.91) or in-hospital survival [62].

In a multi-centre, open-label, phase 3b RCT in VKA-treated patients needing urgent surgical or invasive procedures, effective haemostasis and INR reduction were achieved in more patients from the 4F-PCC group compared to the plasma group, demonstrating both non-inferiority and superiority of 4F-PCC over plasma [63]. The safety profile was similar, but 4F-PCC was associated with fewer fluid overload events. In a meta-analysis of five RCTs and eight observational studies comparing FFP and PCCs for warfarin reversal, PCC use was associated with a significant reduction in all-cause mortality, more rapid INR reduction, and less volume overload without an increased risk of thromboembolic events [64]. In another study, almost 20% of patients who received FFP for warfarin reversal developed pulmonary complications, primarily TACO, and this risk increased with >3 units of FFP administered [65]. A retrospective analysis of data from two prospective phase 3b RCTs confirmed the superiority of PCC compared to FFP for rapid reversal of AVKs (85.9% versus 39.1% of patients had an INR ≤ 1.5 at 30 min after administration of the reversal agent) and demonstrated that, regardless of the post-infusion INR value, all vitamin K dependent factor levels exceeded 50% activity in the PCC group compared to mean activity levels for factors II and X < 50% at all time points assessed within 3 h after starting the infusion of plasma [66].

Furthermore, in a post hoc analysis which evaluated the subgroup of patients undergoing urgent orthopaedic surgery, the use of 4F-PCC compared to plasma was associated with more rapid INR reduction to <1.3 and restoration of vitamin K-dependent factors, and also facilitated earlier surgery (median time to surgery 5.8 vs. 19.2 h, respectively) [67].

Considering the evidence, recent guidelines recommend the use of PCC over plasma for AVK reversal [42,43,50,60], these recommendations being based on the four factor formulation. In VKA-treated patients undergoing an emergency moderate-to-high bleeding-risk procedure, INR must be measured on the patient’s admission to the hospital and 4F-PCC given to reverse VKA anticoagulant effects (at an initial dose of 25 IU FIX/kg at an INR of 4) [42]. Plasma is used for AVK reversal in emergency situations where PCCs are not available [6,7]. However, a recent retrospective, multi-centre study showed that plasma is still frequently used to reverse VKAs [68].

Given the mechanisms of action of the direct oral anticoagulants (DOAC), plasma has minimal impact on activity level of coagulation factors, and patients should receive specific antidotes or PCC for reversal [69]. The role of PCC in the reversal of direct oral anticoagulants differs from its role in vitamin K antagonist reversal. Vitamin K antagonists deplete the circulating vitamin K-dependent factors, which PCC replaces; the direct oral anticoagulants inhibit thrombin or activated factor X, which remain present at normal concentrations. Therefore, reversal depends not on replacing a deficiency but on raising the concentration of substrate sufficiently to overcome the inhibitor, which requires a concentrated product. Plasma contains coagulation factors at physiological concentration, so the volume needed to achieve any meaningful increase is prohibitive, and it does not appear among the agents recommended for this indication. Current guidance recommends that PCC or andexanet alfa be considered in patients on factor Xa inhibitors presenting with severe bleeding, noting that the superiority of one agent over the other has not been demonstrated (1C), and that idarucizumab be considered for dabigatran-associated severe bleeding (1C), with PCC or activated PCC suggested where idarucizumab is unavailable (2C) [69].

## 7. Discussion

Prophylactic plasma transfusion rests on three assumptions: that an abnormal coagulation test predicts bleeding, that plasma will correct it, and that correction reduces bleeding. None is supported. Conventional coagulation tests do not predict procedural bleeding [22,41]. At the mildly elevated INR values that prompt most transfusions, plasma produces little change, because the relationship between factor concentration and INR is non-linear and the INR of the transfused plasma is itself close to that range [12,28].

Whether correcting the INR reduces bleeding has never been demonstrated.

The risks are better established than the benefits. Besides circulatory overload, transfusion-related acute lung injury, and allergic reactions, plasma has been associated in surgical and critically ill patients with venous thromboembolism, higher red cell requirements, reoperation for bleeding, and fewer hospital- and ICU-free days, in proportion to the volume transfused [31,36,45]. Haemodilution and the anticoagulant proteins contained in plasma are plausible explanations.

Despite two decades of consistent evidence, audits show that a third of plasma transfusions follow an INR below 1.8, that the indication is frequently unrecorded, and that practice varies widely between centres and between specialties [3,4]. Changing this requires a different clinical question. A bleeding history is more informative than a coagulation screen; a treatable cause such as vitamin K deficiency or anticoagulant effect should be addressed directly; a lower-risk technique should be used where one exists [24,41]. Where correction is genuinely needed, four-factor PCC, specific antidotes, and fibrinogen concentrate are more effective than plasma and require far less volume.

Patient blood management provides the structure for implementation. In 1174 cardiac surgical patients, a PBM programme reduced perioperative FFP transfusion from 22.9% to 14.2% without any increase in bleeding or adverse outcomes [70]. Audit with feedback, decision support at the point of ordering and better transfusion teaching are needed alongside guidelines [5]. This review is narrative, and the literature it draws on is heterogeneous, largely observational, and graded low or very low certainty throughout. Whether any subgroup benefits, and whether viscoelastic testing could identify one, remains unanswered; randomised trials comparing prophylactic plasma with no prophylaxis are needed and are now ethically feasible.

## 8. Conclusions

In summary, evidence does not support the routine prophylactic use of plasma to correct abnormal coagulation tests in non-bleeding patients undergoing surgery or invasive procedures, including those on AVK anticoagulation. Plasma should not be used solely to normalise mildly prolonged CCTs. For urgent AVK reversal, PCC is preferred, while plasma should generally be reserved for situations in which specific concentrates are unavailable. The expected benefit of plasma transfusion should always be weighed against its potential adverse effects.

## Figures and Tables

**Figure 1:**
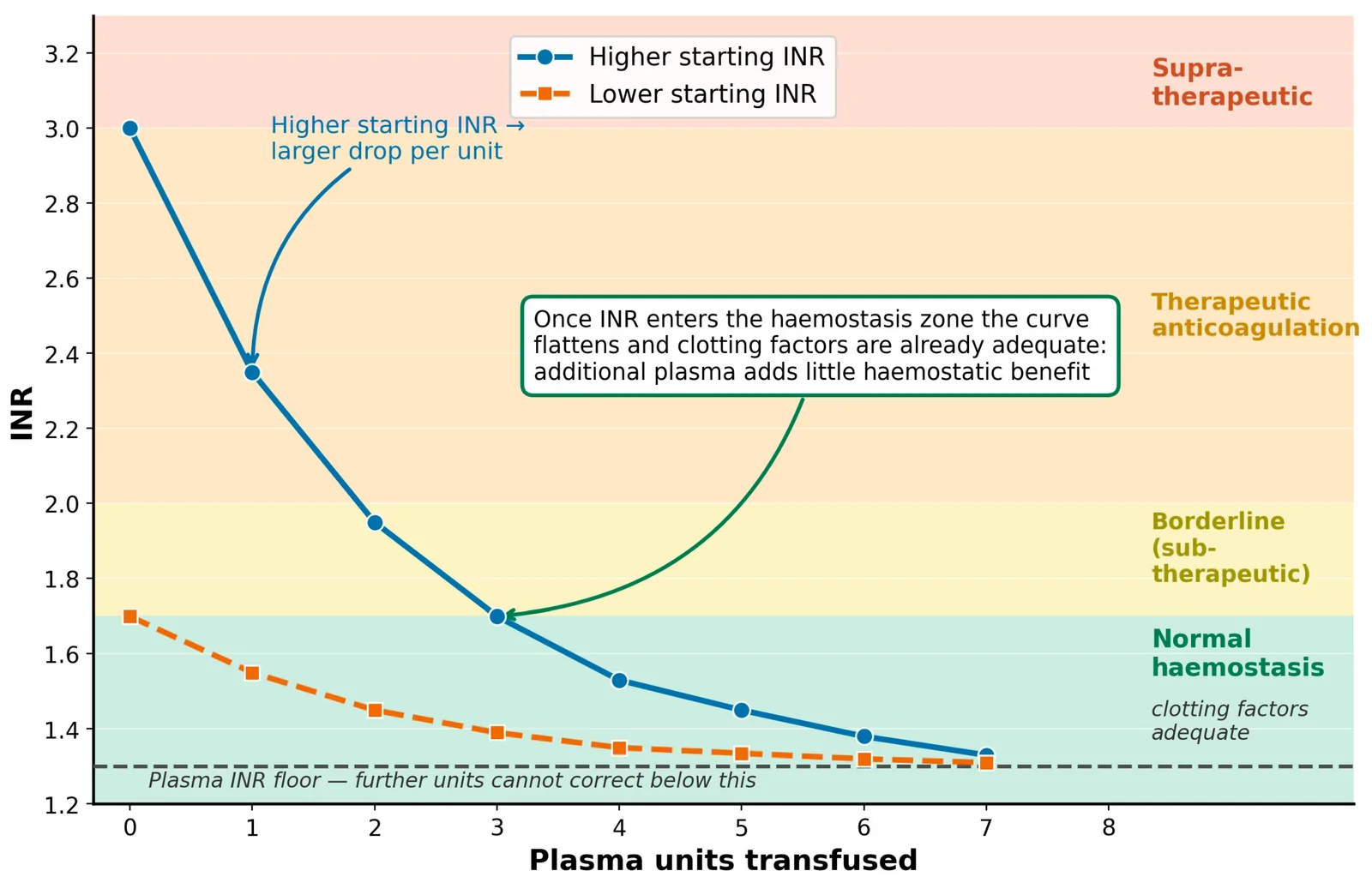
Modelled effect of plasma transfusion on the international normalised ratio (INR) as a function of the number of plasma units transfused, for patients with a higher versus a lower pre-transfusion INR. The higher the starting INR, the greater the reduction achieved per unit; once the INR reaches the normal-haemostasis zone, the curve flattens and clotting factor levels are already adequate, so additional plasma provides little further correction. Because transfused plasma itself has an INR of approximately 1.3, additional units cannot lower the INR below this floor.

**Table 1:** Characteristics of therapeutic plasma preparations (from references [1,6,7,8,9,11]).

Characteristics/Preparations	FFP	SDP	Liquid Plasma	Lyophilised
Source of plasma	Human blood (male only, in some countries)	Pooled from hundreds of units of donated plasma that have been tested for infectious diseases and non-enveloped viruses.	Human whole blood unit before expiration time	Single donor plasma from which the liquid component of plasma was removed under vacuum pressure, to make a powdered preparation
Storage	Rapidly frozen to ≤−25 °C (Europe/UK); ≤−18 °C (FDA/AABB)	Frozen to <−30 °C	Never frozen Stored at 1–6 °C	2–25 °C
Shelf life	1 year frozen; 24 h for thawed plasma	3 years 12 h after thawing	Depends on the preservative solution for the whole blood from which is prepared; up to 5 days after expiration time of the original whole blood unit	15 months
Volume	200–275 mL As high as 600 mL for apheresis units	200 mL	200–300 mL	200 mL or a more concentrated product
Content	All human plasma proteins, coagulation factors, natural and anticoagulants. No RBC or platelets.	Not clinically significant lower concentration of coagulation factors. Highly diluted antibodies from a specific donor.	Same as FFP, although factors V and VIII may decline substantially after prolonged storage. Sufficient quantities for urgent situations, especially trauma transfusion. May contain residual platelets, platelet microparticles, and viable lymphocytes	Content of coagulation factors similar to FFP
Processed	Leukodepletion and pathogen reduction (optional).	Specific pathogen reduction (solvent detergent).	May be irradiated to eliminate the risk of transfusion associated graft versus host disease.	
ABO compatibility	Yes, Rh not necessary	ABO group specific	Yes	Universal compatibility
Preferred clinical use	Multifactorial coagulopathy with active bleeding; therapeutic plasma exchange, including TTP; congenital deficiencies for which no specific concentrate exists (e.g., factor V, factor XI); VKA reversal when PCC is unavailable	Therapeutic plasma exchange; settings prioritizing standardized factor content and pathogen safety. Protein S and α2-antiplasmin are significantly reduced, warranting caution where depletion of natural anticoagulants is a concern	Massive haemorrhage protocols and trauma resuscitation, where immediate availability without thawing is decisive	Pre-hospital, military and remote settings, and early trauma-induced coagulopathy, where ambient-temperature storage and reconstitution within minutes are decisive

FFP—fresh frozen plasma; RBCs—red blood cells; SDP—solvent detergent plasma.

**Table 2:** Selected evidence on prophylactic and peri-interventional plasma transfusion [19,26,27,28,29,30,31,32,33,34,35,36,37,38,39].

Study	Design	Population	Plasma Dose	Laboratory Effect	Clinical/Bleeding Outcome
***Studies reporting laboratory endpoints***					
Holland 2006 [26]	Retrospective analysis with derived regression	Patients receiving plasma across clinical settings	Varied	INR change per unit = 0.37 (pre-transfusion INR)—0.47, r^2^ = 0.82; only 50% of patients with an INR of 1.7 showed a significant change; minimally prolonged INRs fell with treatment of the underlying disease alone, and FFP added nothing further	Not assessed
Abdel-Wahab 2006 [27]	Prospective audit, 121 patients, 324 units	Mild coagulation abnormalities (INR 1.1–1.85)	Varied	PT-INR normalized in 0.8% of patients	Pre-transfusion PT-INR, aPTT, platelet count and creatinine showed no correlation with red cell loss; clinical benefit not directly assessed
Chowdary 2004 [19]	Small non-randomised sequential-dose comparison, 22 patients	Critically ill, prolonged prothrombin time	12.2 vs. 33.5 mL/kg	Dose-dependent relationship: significantly higher increments in fibrinogen and factors V, VII, IX, X, and XII with the higher dose, with substantial variation between individual factors	Not assessed
Pham 2016 [30]	Model derivation and validation against multicentre trial data	Critically ill non-bleeding patients with coagulopathy receiving prophylactic plasma	Varied	Model predicted immediate post-transfusion INR; 83% of predictions within the prespecified acceptable range (|Δ| ≤ 15%), with no proportional difference or systematic bias	Not assessed
Warner 2018 [28]	Retrospective cohort, 6779 patients	85% perioperative; 20% prophylactic before a procedure	Median 2 units; 20% received ≥ 10 mL/kg	Median pre-transfusion INR 1.9, median fall 0.4; complete normalisation (≤1.1) in 12% and partial (≤1.5) in 61%; fall of 2.4 if INR > 3.5 vs. 0.3 if <2.5; no further gain above 10–15 mL/kg	Not assessed
Kurucz 2025 [32]	Prospective international multicentre observational study, sepsis sub-analysis, 799 patients	Septic patients in intensive care; plasma given to 108 (13–14%)	Median 1 transfusion event per patient (IQR 1–2); 2 units per event (IQR 1–3); 3 total units per patient (IQR 2–5) *	INR 1.6 (IQR 1.3–2.4) before and 1.5 (1.2–2.0) after transfusion, median decrease 0.2 (0.0–0.6); an INR target was stated for 59% of events (median target 1.3)	Not assessed
***Studies reporting laboratory and clinical endpoints***					
Müller 2015 [17]	Randomised controlled trial, 81 patients, stopped early	Non-bleeding critically ill, INR 1.5–3.0, before central venous catheter, tracheostomy, chest tube, or abscess drainage	12 mL/kg vs. none	INR reduced to <1.5 in 54% of transfused patients	No difference in bleeding (1 major and 13 minor events in total) or lung injury scores; underpowered for bleeding
Carson 2021 [33]	Pilot randomised controlled trial, 57 patients (34, 59.6%, with cirrhosis)	Hospitalised patients, mean INR 1.92, procedures outside the operating room	10–15 mL/kg vs. none	Post-procedure INR < 1.5 in 10 plasma recipients vs. 1 control (reported as 38.5% and 3.6%; *p* < 0.01) †; mean INR change on the day of the procedure 0.24 (SD 0.26) vs. 0.00 (SD 0.15)	No significant difference in haemoglobin change: −0.6 (SD 1.0) vs. −0.4 (SD 0.6) g/dL; *p* = 0.29
Rocha 2020 [34]	Randomised controlled trial, 57 patients, three protocols, stopped early	Critically ill cirrhotic patients before central venous catheterisation	Protocol-dependent blood component transfusion	Not an endpoint	Transfused before catheterisation: 15.8% restrictive, 68.4% thromboelastometry-guided, 73.7% coagulogram-guided; thromboelastometry guidance no better than coagulogram (OR 0.77, *p* = 0.93). No major bleeding recorded and not powered for bleeding safety; restrictive protocol cost-saving
***Studies reporting clinical outcomes***					
Warner 2017 [35]	Retrospective propensity-matched cohort, 27 561 patients	Critically ill patients; plasma in 2472 (9.0%), prophylactic in 1105 (44.7%)	Median 2 units (IQR 1–2), approximately 7 mL/kg	Not assessed	Plasma associated with more red cell transfusion in the ensuing 24 h (OR 4.3, 95% CI 3.3–5.7) and fewer hospital-free days (−11.0%); no significant difference in ICU-free days or mortality
Jia 2016 [36]	Propensity-matched cohort study, 1234 patients	Non-cardiac surgery, preoperative INR ≥ 1.5; plasma in 139 (11.3%)	Not stated	Not assessed	Unadjusted: more WHO grade 3 perioperative bleeding (52.5% vs. 32.0%), intraoperative red cell transfusion (40.3% vs. 24.5%), and reoperation for bleeding (11.5% vs. 4.5%). After matching, WHO grade 3 bleeding remained more frequent (52% vs. 40%; OR 1.75, 95% CI 1.09–2.81; *p* = 0.021) and ICU admission was more frequent (OR 1.59, 95% CI 1.01–2.48), whereas intraoperative red cell transfusion (OR 1.46, 95% CI 0.90–2.36) and reoperation (OR 1.70, 95% CI 0.78–3.69) were not significant
Warner 2019 [31]	Retrospective cohort, 3393 patients	Intraoperative plasma transfusion	Median 2 units	Higher plasma volumes produced larger INR reductions; greater INR reduction separately associated with better outcomes	Higher plasma volume associated with more red cell transfusion and fewer hospital- and ICU-free days per 1 mL/kg
Desborough 2015 [37]	Cochrane meta-analysis, 15 RCTs (14 prophylactic), 755 patients	Cardiovascular surgery, predominantly elective cardiac surgery with cardiopulmonary bypass	Varied between trials	Not assessed	No reduction in perioperative blood loss; FFP inferior to control for preventing red cell transfusion (Peto OR 2.57, 95% CI 1.30–5.08); no difference in return to theater (Peto OR 0.81)
Fu 2024 [38]	Retrospective propensity-matched cohort (MIMIC-IV), 693 patients, 233 per group	Decompensated cirrhosis with significant coagulopathy in intensive care	Variable; no standardised regimen reported	During 14-day follow-up, INR, aPTT and total bilirubin were higher in plasma recipients	No significant adjusted association with in-hospital (HR 1.30), 30-day (HR 1.08) or 90-day (HR 1.03) mortality; plasma associated with liver (OR 3.00), kidney (OR 1.90), coagulation (OR 2.55), respiratory (OR 1.76) and circulatory (OR 2.15) failure, and with longer ICU and hospital stay. Each additional 1 mL/kg was associated with higher odds of liver, kidney and circulatory failure
Huber 2019 [39]	Cochrane systematic review, 5 trials included; findings based on 4 trials (234 participants)	Non-cardiac surgery and invasive procedures, without inherited bleeding disorders or anticoagulant use	Varied between trials	Not assessed	No demonstrated difference in mortality, bleeding, transfusion requirement or transfusion-related adverse events; all outcomes very low-quality evidence

* The source reports a median of 3 units per transfusion event in the text and 2 units per event in Table 2; the figures are reproduced as published. † Counts and percentages are reproduced as published; the reported proportions do not correspond exactly to the stated denominators. INR correction is a surrogate outcome and does not itself indicate reduced bleeding risk. Findings from retrospective cohorts are associations and do not establish that plasma caused the reported clinical outcomes. aPTT—activated partial thromboplastin time; CI—confidence interval; FFP—fresh frozen plasma; HR—hazard ratio; ICU—intensive care unit; INR—international normalised ratio; IQR—interquartile range; MIMIC—Medical Information Mart for Intensive Care; OR—odds ratio; PT—prothrombin time; RCT—randomised controlled trial; SD—standard deviation.

**Table 3:** Classification of procedures by bleeding risk (guideline recommendations from [24]).

	Low Bleeding Risk	High Bleeding Risk *
**Endoscopic**	Diagnostic procedures, endoscopic variceal ligation, transoesophageal echocardiography	Bronchoscopy with biopsy, colonoscopy with polypectomy, endoscopic retrograde cholangiopancreatography with sphincterotomy
**Percutaneous**	Paracentesis, thoracocentesis	Percutaneous liver biopsy, tunnelled ascitic or pleural drain placement, cranial and spinal surgery †
**Vascular**	Peripheral and central venous catheterisation, transjugular liver biopsy	Transjugular intrahepatic portosystemic shunt, transcatheter arterial chemoembolisation
**Other**	Dental procedures including extractions, skin biopsy	Intraocular procedures †

* Major bleeding rate above 1.5%, or minor bleeding associated with a high risk of significant organ damage or death. † Very high risk procedures. A concordant two-tier classification is given in {Lester 2024 [41]}, which places basic venous interventions and superficial biopsies at low risk, and solid organ puncture, embolisation, and transjugular intrahepatic portosystemic shunt at moderate to high risk.

**Table 4:** Pre-procedural coagulation test thresholds in current guidelines and consensus statements [13,24,41,43,44,47,50,52].

Guideline	Scope	Stated Pre-Procedural Threshold	Strength/Certainty
BSH 2024 [41]	Invasive procedures, general	None. Routine coagulation screening not recommended, as it does not indicate bleeding risk; routine plasma to correct abnormal results not recommended	1C
BSH 2018 [13]	FFP and cryoprecipitate outside major bleeding	None. No evidence for prophylactic FFP pre-procedure in non-bleeding patients; effect of commonly used doses very limited when the PT ratio or INR is 1.5–1.9	2C
ESICM 2020 [47]	Non-bleeding critically ill	None. Against prophylactic plasma in patients with coagulopathy, and against prophylactic plasma before invasive bedside procedures	Conditional, very low certainty
ACCP 2025 [44]	Critically ill adults	None for central venous catheter or arterial line insertion, bedside thoracentesis or paracentesis, flexible bronchoscopy without biopsy, or endoscopy in portal hypertension-related bleeding. FFP suggested to target INR 1.5–2.0 before lumbar puncture	Conditional, very low certainty
AoA 2025 [50]	Blood components and alternatives	None. No good evidence for prophylactic FFP before low-risk invasive procedures in critically ill patients; prophylactic FFP not recommended in elective cardiac surgery	[insert]
EACTS/EACTAIC 2024 [43]	Adult cardiac surgery	None. PT and aPTT were not found to be associated with the amount of perioperative bleeding or transfusion requirements	[insert]
ISTH SSC 2022 [24]; AASLD 2021 [52]	Cirrhosis, periprocedural	None. PT/INR should not be routinely evaluated to predict bleeding risk, even in critically ill patients; against prophylactic correction of abnormal coagulation parameters in the absence of vitamin K antagonist use. Of five societies compared, only SIR 2019 names a value (INR > 2.5), advising vitamin K rather than FFP or PCC	Weak guidance statement
EASL 2017 [53]	Acute liver failure	None routinely. Use of FFP and other coagulation factors not supported, and should be limited to specific situations such as insertion of ICP monitors or active bleeding	Evidence level II-3, grade 1

Only two numerical values appear across these documents: the conditional suggestion of an INR of 1.5–2.0 before lumbar puncture in critically ill patients, and an INR above 2.5 in cirrhosis, for which vitamin K rather than plasma is advised. No guideline recommends prophylactic plasma transfusion for an INR below 1.8 in a non-bleeding patient. AASLD—American Association for the Study of Liver Diseases; ACCP—American College of Chest Physicians; AoA—Association of Anaesthetists; aPTT—activated partial thromboplastin time; BSH—British Society for Haematology; EACTS/EACTAIC—European Association for Cardio-Thoracic Surgery/European Association of Cardiothoracic Anaesthesiology and Intensive Care; EASL—European Association for the Study of the Liver; ESICM—European Society of Intensive Care Medicine; FFP—fresh frozen plasma; ICP—intracranial pressure; INR—international normalized ratio; ISTH SSC—International Society on Thrombosis and Haemostasis Scientific and Standardisation Committee; PCC—prothrombin complex concentrate; PT—prothrombin time; SIR—Society of Interventional Radiology.

## Data Availability

No new data were created or analysed in this study. Data sharing is not applicable to this article.

## Abbreviations

The following abbreviations are used in this manuscript:

aPTT	activated partial thromboplastin time
AVKs	vitamin K antagonists
DOAC	direct oral anticoagulants
FFP	fresh frozen plasma
ICU	intensive care unit
INR	international normalised ratio
PBM	patient blood management
PCC	prothrombin complex concentrate
PT	prothrombin time
RBC	red blood cell
RCT	randomised controlled trial
TACO	transfusion-associated circulatory overload
TRALI	transfusion-related lung injury
VET	viscoelastic tests
